# Malignant Glaucoma After Trabeculectomy—Diagnosis and Treatment Options: A Case Report

**DOI:** 10.3390/reports9020102

**Published:** 2026-03-27

**Authors:** Ada Sterczewska, Adrian Smędowski, Justyna Sierocka-Stępień, Dorota Wyględowska-Promieńska, Mariola Dorecka

**Affiliations:** 1 Department of Ophthalmology, Prof. K. Gibinski University Clinical Centre, Medical University of Silesia in Katowice, 40-514 Katowice, Poland; 2 Department of Ophthalmology, Faculty of Medical Sciences in Katowice, Medical University of Silesia in Katowice, 40-514 Katowice, Poland

**Keywords:** malignant glaucoma, trabeculectomy, irido-zonulectomy with pars plana vitrectomy

## Abstract

**Background and Clinical Significance:** Malignant glaucoma, also described as aqueous misdirection syndrome, most commonly occurs following trabeculectomy in patients with primary angle-closure glaucoma and a shallow anterior chamber. Management aims to restore normal anterior segment anatomy, re-establish aqueous flow from the posterior to the anterior chamber, and achieve adequate intraocular pressure control. This report presents a case of malignant glaucoma developing after trabeculectomy, with emphasis on current diagnostic and therapeutic approaches. **Case Presentation:** A patient with primary angle-closure glaucoma, pseudophakia of the right eye, and a patent laser peripheral iridotomy, receiving maximal tolerated topical antiglaucoma therapy, was referred to the Department of Ophthalmology due to uncontrolled intraocular pressure. The patient was scheduled for trabeculectomy of the right eye. In the immediate postoperative period, intraocular pressure was adequately controlled. However, on postoperative day five, a significant elevation in intraocular pressure was observed, accompanied by persistent shallowing of the anterior chamber. Topical and systemic hypotensive therapy, posterior capsulotomy and hyaloidotomy were performed without improvement of the local condition. The patient was qualified for irido-zonulectomy with pars plana vitrectomy. Following surgical intervention, normalization of intraocular pressure was achieved, and the anatomy of the anterior chamber was restored. **Conclusions:** Malignant glaucoma remains a challenging postoperative complication and is frequently refractory to conservative pharmacological and laser-based interventions. Early recognition and prompt surgical management, particularly irido-zonulectomy combined with pars plana vitrectomy, significantly increase the likelihood of anatomical and functional success.

## 1. Introduction and Clinical Significance

Historically, malignant glaucoma (MG) was first described in 1868 by Albrecht von Graefe [1]. It is also referred to as aqueous misdirection syndrome, ciliolenticular block glaucoma, or iridociliary–vitreous block glaucoma [2]. It is an aggressive, severe, and difficult-to-treat course, and this explains why the condition is termed MG. This disease entity accounts for fewer than 2% of all glaucoma cases. MG occurs more frequently in elderly patients, women, and individuals with hyperopia, a narrow ciliary sulcus or iridocorneal angle, plateau iris configuration, or a history of miotics use [3,4]. It most commonly develops following intraocular surgery, particularly trabeculectomy, but has also been reported after cataract extraction, glaucoma drainage device implantation and pars plana vitrectomy, with an estimated incidence of 2–4% in susceptible populations [5,6]. The onset of MG may range from several days to several years after surgery [7]. Differential diagnosis of MG includes pupillary block glaucoma, serous choroidal detachment, suprachoroidal hemorrhage, plateau iris syndrome, phacomorphic glaucoma, anterior lens subluxation, and postoperative overfiltration with hypotony and flat anterior chamber. The precise pathophysiology of MG remains incompletely understood, and several mechanisms have been proposed. Shaffer and Hoskins suggested that posterior misdirection of aqueous humor leads to its accumulation behind the vitreous body, resulting in anterior displacement of the lens–iris diaphragm [8]. Chandler proposed that zonular laxity combined with increased vitreous pressure causes forward movement of the crystalline lens, creating a self-perpetuating cycle in which rising posterior segment pressure further displaces the lens anteriorly [4]. Quigley et al. hypothesized that choroidal expansion, followed by compensatory aqueous flow along a posterior-to-anterior pressure gradient, contributes to progressive shallowing of the anterior chamber (AC) [9]. Epstein et al. [10] postulated that MG results from a diminished permeability of the vitreous body (VB) to the anterior passage of aqueous humor into the AC. Clinically, MG is characterized by shallowing of the AC in the presence of elevated intraocular pressure (IOP), despite a patent peripheral iridotomy. MG develops despite pseudophakia and prior LPI because its mechanism is not primarily a pupillary block, which is treated using the above methods. Patients may present with ocular pain, blurred vision and a myopic shift due to anterior displacement of the lens–iris diaphragm. Anterior segment optical coherence tomography (AS-OCT) provides valuable diagnostic information by demonstrating reduced AC depth and anterior rotation of the ciliary body, supporting the diagnosis of aqueous misdirection [11]. Management aims to lower IOP and restore normal anterior segment anatomy by re-establishing physiological aqueous humor flow. Treatment strategies include intensive pharmacotherapy (cycloplegics, hyperosmotic agents), laser interventions (e.g., posterior capsulotomy and anterior hyaloidotomy in pseudophakic eyes), and definitive surgical approaches [12,13]. The aim of this study was to present and analyze a case of malignant glaucoma following trabeculectomy in a patient with primary angle-closure glaucoma (PACG), managed using a stepwise approach that included pharmacological, laser, and ultimately surgical treatment. We review the underlying pathophysiological mechanisms and diagnostic considerations of MG and summarize current therapeutic options based on the literature. Particular emphasis is placed on the high efficacy of irido-zonulectomy combined with pars plana vitrectomy (IZ with PPV) as a definitive surgical intervention for refractory malignant glaucoma.

## 2. Case Presentation

**Figure 1:**
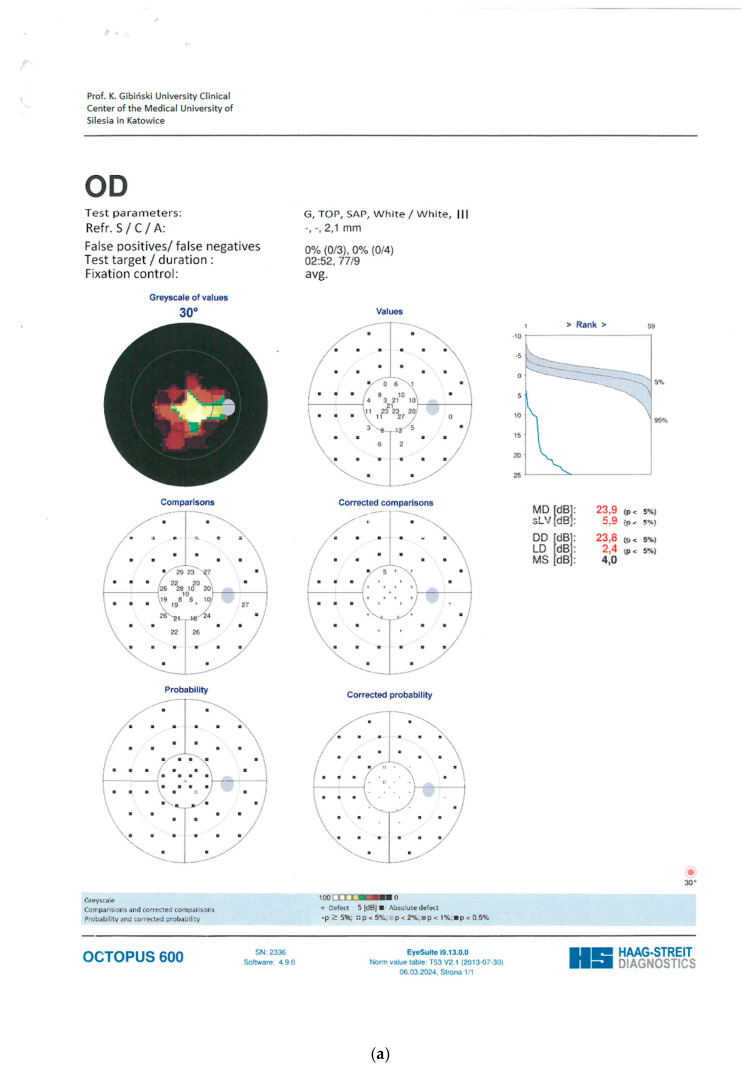

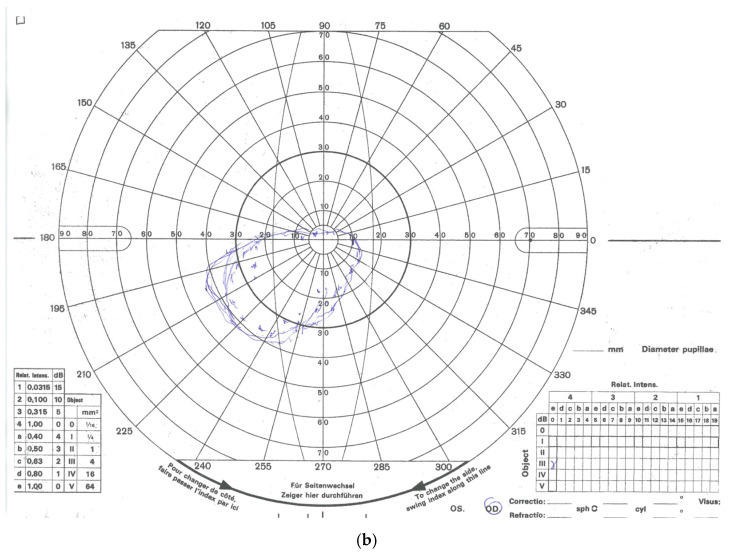
(**a**) The static perimetry of the RE before the trabeculectomy. (**b**) The kinetic perimetry of the RE before the trabeculectomy.

**Figure 2:**
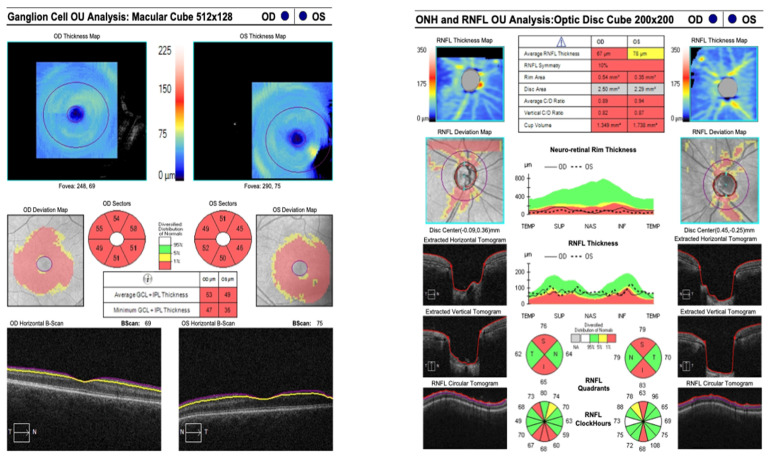
SOCT of both eyes before the trabeculectomy.

A forty-two-year-old female patient was referred to the Department of Ophthalmology with a diagnosis of primary angle-closure glaucoma (PACG). She had previously undergone bilateral laser peripheral iridotomy (LPI). At presentation, best-corrected visual acuity (BCVA) in the right eye (RE) was 0.8 cum correctione (cc) −0.75 Diopter Sphere (Dsph), whereas the left eye (LE) was limited to light perception. Intraocular pressure (IOP), measured by Goldmann applanation tonometry (GAT), was 35 mmHg in the RE and 20 mmHg in the LE on maximum topical pharmacotherapy: brimonidine three times daily, dorzolamide + timolol twice daily, latanoprost once daily. The patient had previously undergone micropulse transscleral cyclophotocoagulation in the RE. The RE was pseudophakic, with implantation of a Rayner RayOne Aspheric +28.50 D intraocular lens. Axial length measured 20.7 mm. Slit-lamp examination of the anterior segment in the RE demonstrated a patent peripheral iridotomy, a shallow anterior chamber, and a well-centered posterior chamber intraocular lens in situ. Funduscopy of the RE revealed advanced glaucomatous optic neuropathy. Gonioscopy demonstrated extensive peripheral anterior synechiae involving the inferior, nasal, and temporal quadrants. The iridocorneal angle was closed in these sectors but could be dynamically opened superiorly. Perimetry and kinetic perimetry of the RE demonstrated concentric visual field constriction; the mean deviation (MD) was 23.9 dB (Figure 1). Spectral-domain optical coherence tomography (SOCT) of the posterior segment revealed marked thinning of the ganglion cell complex and advanced glaucomatous optic neuropathy, with a cup-to-disk ratio (c/d) of 0.89 (Figure 2).

**Figure 3:**
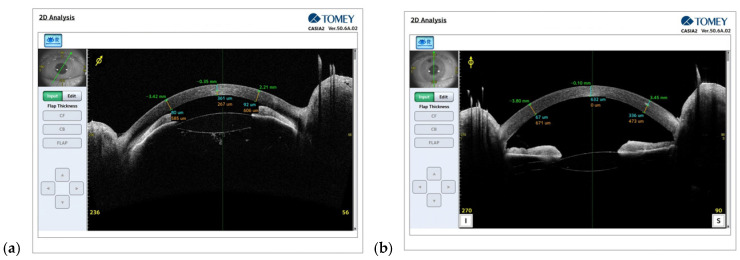
(**a**) AS-OCT of the RE 8 days after trabeculectomy. (**b**) AS-OCT of the RE 3 weeks after irido-zonulo with PPV.

The patient was scheduled for trabeculectomy with intraoperative application of mitomycin C, 0.2 mg/mL, which was administered for 2 min using sponge application subconjunctivally and sub-Tenon’s in the RE. A superior fornix-based conjunctival flap was fashioned, followed by the creation of a 4 × 4 mm half-thickness rectangular scleral flap. A peripheral iridectomy was performed. The scleral flap was secured with two releasable 10-0 nylon sutures. On postoperative day 1, BCVA in the RE was 0.6, and IOP (GAT) in the RE was 14 mmHg. AC was of moderate depth, and a functioning filtering bleb was present. Seidel testing was negative, with no evidence of wound leakage. By postoperative day 5, BCVA had declined to 0.02, IOP (GAT) had increased to 40 mmHg, and slit-lamp examination revealed a markedly shallow AC. Intensive medical therapy was initiated, including topical tropicamide 1% three times daily, timolol twice daily, brimonidine three times daily, and dorzolamide three times daily, in addition to systemic acetazolamide 250 mg and intravenous 20% mannitol. On postoperative day 6, owing to persistently elevated IOP and a lack of anatomical improvement, Nd:YAG laser capsulotomy and anterior hyaloidotomy were performed. However, by postoperative day 8, BCVA in the RE was 0.06 cc −4.0 Dsph, and IOP remained elevated at 32 mmHg. B-scan ultrasonography of the RE revealed no posterior segment pathology. Anterior segment optical coherence tomography (AS-OCT) demonstrated extreme shallowing of the AC (Figure 3a). Based on the clinical and imaging findings, a diagnosis of malignant glaucoma was established. The best solution with the lowest recurrence rate in comparison to other surgeries was IZ with PPV. The patient subsequently underwent IZ with PPV and intravitreal 20% SF_6_ gas tamponade in the RE with partial iris, posterior capsule, zonular fibers and vitreous removal. On postoperative day 4 following this intervention, BCVA was limited to hand movements, with a residual intraocular gas bubble visible in the vitreous cavity (VC). IOP (GAT) was 16 mmHg. The patient was discharged with topical levofloxacin five times daily, dexamethasone 0.1% five times daily, and tropicamide 1% three times daily with face-down postoperative positioning for 24 h, followed by upright positioning for 3 days. At 3 weeks following IZ with PPV, BCVA in the RE had improved to 0.2, and IOP (GAT) was 20 mmHg. AS-OCT confirmed reformation of the AC and restoration of normal anterior segment anatomy (Figure 3b).

**Figure 4:**
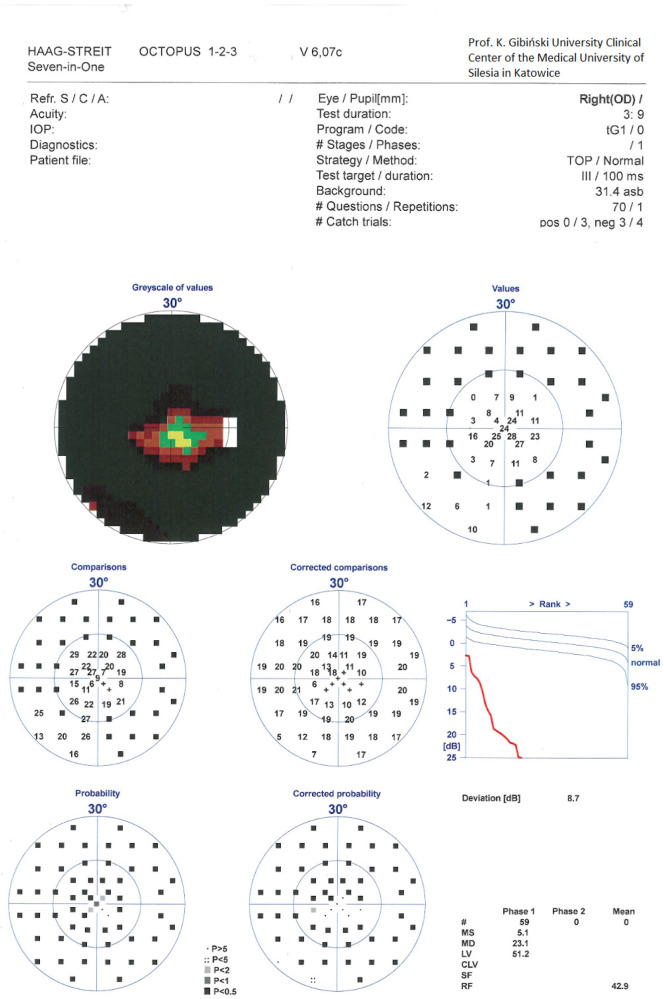
The static perimetry of the RE a year after irido-zonulectomy with PPV.

**Figure 5:**
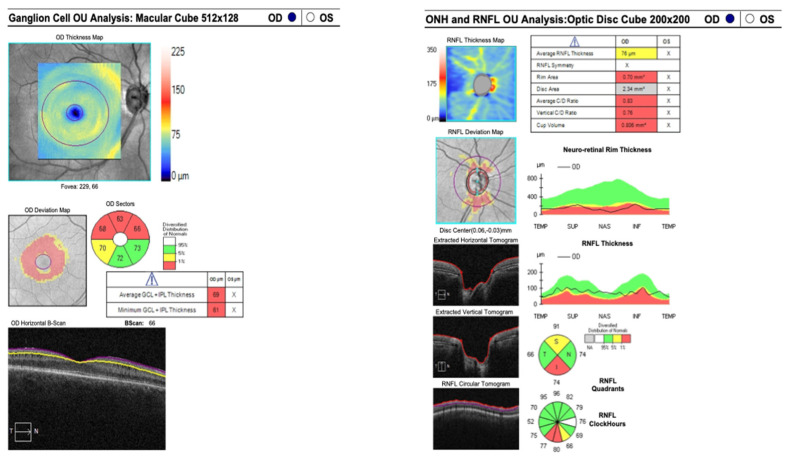
SOCT of the RE 12 months after irido-zonulectomy with PPV.

Postoperatively, topical therapy in the RE was continued, with a fixed combination of dorzolamide/timolol administered twice daily. At 1 month following IZ with PPV, BCVA in the RE improved to 0.5, and IOP (GAT) was 17 mmHg. At 5 months postoperatively, BCVA further improved to 0.7 cc −1.5 Cylinder (Cyl) axis (ax) 160° and IOP (GAT) remained stable at 17 mmHg. At 12 months after IZ with PPV, BCVA was maintained at 0.7 with the same refractive correction, and IOP (GAT) was 17 mmHg. Slit-lamp examination of the anterior segment of the RE demonstrated restoration and maintenance of normal AC anatomy. Funduscopic evaluation revealed features consistent with advanced glaucomatous optic neuropathy. Perimetry of the RE showed persistent concentric visual field constriction; MD was 23.1 dB (Figure 4). SOCT of the posterior segment of the RE demonstrated significant ganglion cell complex loss and advanced glaucomatous structural damage, with c/d of 0.83 (Figure 5). Importantly, there was no clear evidence of structural or functional progression of glaucomatous neuropathy during the 12-month follow-up period. Topical therapy with the fixed combination of dorzolamide + timolol twice daily was maintained.

## 3. Discussion

MG remains clinically challenging due to its complex pathophysiology and high recurrence rate. A range of therapeutic strategies has been described, including pharmacologic therapy, laser interventions, and surgical interventions, each demonstrating variable efficacy.

### 3.1. Pharmacologic Therapy

In most cases, treatment is initiated with pharmacologic therapy. Initial pharmacologic management typically includes cycloplegic agents, aqueous suppressants, hyperosmotic agents, and anti-inflammatory therapy. Cycloplegics induce relaxation of the ciliary muscle, resulting in posterior displacement of the lens–iris diaphragm through zonular tightening and widening of the ciliary ring. This mechanism aims to deepen the AC and counteract the anterior displacement characteristic of MG [2,12,13,14]. IOP reduction is achieved with systemic carbonic anhydrase inhibitors (e.g., acetazolamide) and topical aqueous suppressants, including beta-adrenergic antagonists and α2-adrenergic agonists, which decrease aqueous humor production. Hyperosmotic agents reduce vitreous volume, thereby contributing to deepening of the AC and facilitating posterior segment decompression [12]. Despite these measures, medical therapy alone is infrequently curative. Although transient anatomical improvement may occur in approximately 50% of cases, reported relapse rates approach 100%, underscoring the limited long-term efficacy of conservative treatment [15].

### 3.2. Laser Interventions

Laser interventions represent the next therapeutic step: LPI or YAG capsulotomy with hyaloidotomy. LPI may be performed to exclude pupillary block and to establish an alternative pathway for aqueous humor from the posterior to the anterior chamber, thereby reducing the posterior–anterior pressure gradient. While LPI has demonstrated efficacy in selected cases, particularly when a patent iridotomy is absent, its overall success in established MG is limited [16]. In a cohort study by Lincke et al. [17], LPI was performed in four of twelve eyes that developed MG following cataract surgery. In three of these eyes, LPI effectively abolished the posterior–anterior pressure gradient, leading to reformation and deepening of the AC and normalization of IOP. In pseudophakic or aphakic eyes, Nd:YAG posterior capsulotomy combined with anterior hyaloidotomy aims to disrupt the anterior hyaloid face and create direct communication between the posterior segment and the AC, thereby restoring physiologic aqueous flow [12,18]. Shadid et al. [12] suggested that an intact VB is considered a key pathogenic factor in pseudophakic MG, as it may prevent anterior movement of aqueous humor [6]. According to Pożarowska [2], in phakic eyes, this approach carries a substantial risk of crystalline lens damage and is therefore generally avoided [2,19,20].

### 3.3. Surgical Interventions

When conservative measures fail, surgical intervention is indicated. The limited therapeutic efficacy of pharmacologic and laser interventions is consistent with the findings of Jin and Caprioli, who evaluated seventy-three patients (seventy-four eyes) with MG and similarly reported suboptimal treatment response [21]. Fifty-six eyes received pharmacologic therapy, with complete resolution of MG achieved in only two eyes treated with medical management alone. Seven eyes underwent laser intervention, resulting in resolution in five cases. Foreman-Larkin et al. [14] reported that in cases where posterior capsulotomy combined with hyaloidotomy proved ineffective, the subsequent recommended intervention was pars plana vitrectomy (PPV) with surgical removal of the anterior segment of the VB.

PPV, with removal of the anterior vitreous and disruption of the anterior hyaloid face, is widely regarded as the definitive treatment. A critical determinant of surgical success is not only evacuation of misdirected aqueous from the VC but also the creation of a permanent and functional communication between the VC and the AC [2,19,20,22,23,24]. This typically requires removal of multiple anatomical barriers—including the anterior vitreous, posterior capsule, zonular fibers, and often a segment of peripheral iris—to eliminate the abnormal posterior diversion of aqueous humor and to equalize pressure between the anterior and posterior segments [19].

Debrouwere et al. [19] suggested that complete PPV combined with iridectomy, capsulectomy, and zonulectomy is associated with the lowest recurrence rates. Retrospective analyses have demonstrated that the combined technique of vitrectomy–iridectomy–zonulectomy (and phacoemulsification in phakic patients) had the lowest (0%) recurrence rate when compared to vitrectomy (75% of recurrence) or YAG capsulotomy with hyaloidotomy (75% of recurrence).

In pseudophakic eyes, iridectomy–hyaloidotomy–zonulectomy combined with anterior vitrectomy (AV) has similarly shown favorable outcomes. That was confirmed by Rękas et al. [25] in seventeen patients (twenty eyes) with MG. The high effectiveness of combined pars plana antero-central vitrectomy, hyaloido-zonulectomy and iridectomy in MG was also confirmed by the previously cited Jin and Caprioli [21]. Among the fifty-five eyes which had surgical treatment, fifty-two eyes failed pharmacological treatment, and three eyes were treatment naïve. The anatomical success rate was 96.4%. Alternative anterior segment approaches, including zonulo-hyaloido-vitrectomy performed by anterior segment surgeons, have been described, particularly for pseudophakic MG [23,26]. This technique involves the creation of a channel through a peripheral iridectomy, zonule, and posterior capsule to access the anterior vitreous, followed by AV and excision of obstructing structures [2,22,23]. After AV, the vitrectomy device is slowly withdrawn and the capsule, zonule and iris are excised [23,25].

Żarnowski et al. [23] treated ten eyes with MG using a novel surgical technique, an anterior chamber capsulo-hyaloidectomy and AV through the iridectomy. All cases have a relief of aqueous misdirection, AC and post-operative IOP normalization. There were no relapses during follow-up, which lasted 6–18 months. When MG develops in a phakic eye, most investigations recommend performing vitrectomy and cataract extraction simultaneously [24].

In the presence of peripheral anterior synechiae or significant anterior segment fibrosis, additional procedures, such as goniosynechiolysis or implantation of glaucoma drainage devices, may be required in conjunction with PPV [27].

Postoperative complications must also be considered. Cystoid macular edema has been reported by Lincke et al. [17] as one of the most common adverse events following IZ with PPV in eyes with MG after cataract surgery.

### 3.4. Risk of Developing Malignant Glaucoma in the Fellow Eye

Furthermore, patients with MG in one eye are at increased risk of developing the condition in the fellow eye [27]. Prophylactic measures, including laser iridotomy or iridectomy in anatomically predisposed fellow eyes, may be considered. In selected high-risk cases, particularly when advanced MG necessitated PPV in the first eye, prophylactic combined phacoemulsification and vitrectomy in the fellow eye has been proposed.

## 4. Conclusions

Therapeutic success in MG is predicated on two principal objectives: (1) re-establishment of unobstructed aqueous humor flow between the posterior and anterior segments of the eye, thereby eliminating aqueous misdirection, and (2) restoration of normal anterior segment anatomy, characterized by deepening of the AC and sustained reduction in IOP, ideally without the need for long-term polypharmacotherapy or with minimal adjunctive medical therapy. Advances in surgical management—particularly techniques facilitating disruption of the anterior hyaloid face and establishment of a permanent communication between the VC and AC—have substantially improved anatomical and functional outcomes. Concurrent progress in diagnostic imaging, including high-resolution AS-OCT, has enhanced our ability to detect early anatomical alterations and guide timely intervention. Furthermore, improved recognition of high-risk populations and the prompt initiation of appropriate therapy have contributed to a greater likelihood of preserving visual function in patients with MG. Collectively, these developments have led to improved long-term structural stabilization and the maintenance of functional visual parameters in spite of this challenging clinical entity.

## Data Availability

The original data presented in this study are available on reasonable request from the corresponding author. The data are not publicly available due to privacy concerns.

## Abbreviations

The following abbreviations are used in this manuscript:

MG	malignant glaucoma
AC	anterior chamber
VB	vitreous body
IOP	intraocular pressure
OCT	optical coherence tomography
PACG	primary angle-closure glaucoma
IZ with PPV	irido-zonulectomy with pars plana vitrectomy
LPI	laser peripheral iridotomy
BCVA	best corrected visual acuity
RE	right eye
cc	cum correctione (with correction)
Dsph	diopter sphere
LE	left eye
GAT	Goldmann applanation tonometer
MP-TSCPC	micropulse transscleral cyclophotocoagulation
IOL	intraocular lens
AXL	axial length of the eye
MD	mean deviation
SOCT	spectral optical coherence tomography
c/d	cup-to-disk ratio
AS-OCT	anterior segment optical coherence tomography
VC	vitreous cavity
Cyl	cylinder
ax	axis
PPV	pars plana vitrectomy
AV	anterior vitrectomy
